# Impact-Induced Reaction Characteristic and the Enhanced Sensitivity of PTFE/Al/Bi_2_O_3_ Composites

**DOI:** 10.3390/polym11122049

**Published:** 2019-12-10

**Authors:** Ying Yuan, Baoqun Geng, Tao Sun, Qingbo Yu, Haifu Wang

**Affiliations:** State Key Laboratory of Explosion Science and Technology, Beijing Institute of Technology, Beijing 100081, China; 3120185181@bit.edu.cn (Y.Y.); gengbq@bit.edu.cn (B.G.); 3120185143@bit.edu.cn (T.S.); yuqb@bit.edu.cn (Q.Y.)

**Keywords:** PTFE/Al/Bi_2_O_3_, reaction characteristic, impact sensitivity, energy release, reaction mechanism

## Abstract

In this paper, the reaction characteristic of a novel reactive material, which introduced bismuth trioxide (Bi_2_O_3_) into traditional polytetrafluoroethylene/aluminum (PTFE/Al), is studied. The effect of Bi_2_O_3_ with different content and particle size on the reaction behaviors of PTFE/Al/Bi_2_O_3_ are investigated by drop-weight test and X-ray diffractometer (XRD), including impact sensitivity, energy release performance under a certain impact, and reaction mechanism. The experimental results show that the content of Bi_2_O_3_ increased from 0% to 35.616%, the characteristic drop height of impact sensitivity (*H*_50_) of PTFE/Al/Bi_2_O_3_ reactive materials decreased first and then increased, and the minimum *H*_50_ of all types of materials in the experiment is 0.74 times that of PTFE/Al, and the particle size of Bi_2_O_3_ affects the rate of *H*_50_ change with Bi_2_O_3_ content. Besides, with the increase of Bi_2_O_3_ content, both the reaction intensity and duration first increase and then decrease, and there is optimum content of Bi_2_O_3_ maximizing the reaction degree of the PTFE/Al/Bi_2_O_3_. Furthermore, a prediction model for the impact sensitivity of PTFE-based reactive material is developed. The main reaction products include AlF_3_, xBi_2_O_3_·Al_2_O_3_, and Bi.

## 1. Introduction

Reactive materials, a special type of energetic material—including metal/polymer mixture, intermetallic, thermite, hydrides, and matrix material [1,2]—have been widely concerned. Metal/polytetrafluoroethylene (PTFE) as a representative, due to the high energy density and impact-induced reaction characteristics, has broad application prospects.

PTFE, whose chemical formula is –(CF_2_–CF_2_)–, is a complicated semi-crystalline material with great stability and chemical inertness at room temperature [3]. It is noteworthy that PTFE has a special property: the as-polymerized melting temperature is about 341 °C, while the subsequent melting temperature is about 328 °C. PTFE has at least four known phases at different pressures and temperatures. Generally speaking, at the high temperature, PTFE mainly presents as pseudohexagonal or orthorhombic crystals, while at the low temperature, PTFE mainly presents as triclinic or hexagonal crystal [4]. At a higher temperature (more than 380 °C), because of PTFE decomposition, the volatile fluorinated gases are released, which have the ability to react with active metals releasing large amounts of heat [5].

In PTFE-based reactive material, PTFE acts not only as a reductant but also as a binder. It can closely wrap particles through high-temperature sintering, and its internal connection is relatively close. Therefore, PTFE-based energetic materials usually have excellent impact properties. Metals, as oxidants, participate in the reaction and release a large amount of heat. The properties of metals directly affect the energy release performance of reactive materials. A variety of metal/PTFE-based reactive materials, such as PTFE/Al, PTFE/Mg, PTFE/Ti, PTFE/Ni, and PTFE/Ta, were presented, and their reaction mechanism was studied. The study shows that the reaction mechanism of metal/PTFE filled with different metals strongly depends on the metal properties [5].

PTFE/Al reactive materials are especially of concern among researchers because of their excellent reaction performance. Many scholars have conducted a lot of research on PTFE/Al reactive materials’ properties, including mechanical properties and energy release performance. Raftenberg [6] performed Taylor rod tests and Hopkinson pressure bar (SHPB) experiments to study the deformation and compressive mechanical responses of PTFE/Al. Cai [7], based on the simulation and experimental results, investigated the effect of particle size of Al and porosity of W on the mechanical properties of materials from the perspective of force chain. Ames [8] studied the energy release characteristics of PTFE/Al and developed a model that illuminates the connection between energy release and pressure.

Although PTFE/Al has higher strength and density than traditional energetic materials, there are still a lot of problems that need to be solved, including low energy release rate and high reaction threshold, resulting in incomplete reaction. To improve energy release performance, some energetic components were introduced into PTFE/Al, such as Ta [5], Ni [9], CuO [10], MoO_3_ [11], and TiH_2_ [12]. The experiments indicated that the additional components induce additional exothermic reactions except reaction between PTFE and Al. It improves the energy release performance of composite material. Bismuth oxide (Bi_2_O_3_), as a high-density metal oxide (8.9 g·cm^−3^), could undergo an intense redox reaction with Al. Al/Bi_2_O_3_, as a high-energy thermite, is characterized by a fast burning rate, high peak combustion pressure, and a high pressurization rate, compared with other thermites [13]. Because of these particular properties, Al/Bi_2_O_3_ has been added to propellants and percussion primer to improve performance [14]. In this way, the energy release performance of reactive materials can be greatly improved, which has a broad application prospect. However, little published papers have studied the reaction behaviors of PTFE/Al/Bi_2_O_3_ composite.

In this paper, PTFE/Al/Bi_2_O_3_, a novel reactive material, was designed and prepared for the first time. The effect of Bi_2_O_3_ content and particle size on the impact sensitivity and energy release performance of PTFE/Al/Bi_2_O_3_ was studied by drop-weight tests. Based on the experimental results, the reaction mechanism of the material was analyzed from mechanics. In addition, this paper takes PTFE/Al materials with Bi_2_O_3_ as an example to develop a semi-quantitative theoretical model of impact sensitivity of PTFE/Al-based reactive materials. The chemical reaction of PTFE/Al/Bi_2_O_3_ under impact was analyzed by X-ray diffraction (XRD), and the mechanism of Bi_2_O_3_ enhancing the energy release performance of PTFE/Al/Bi_2_O_3_ energetic composite was presented.

## 2. Materials and Methods

### 2.1. Sample Preparation

The raw materials were as follows: PTFE powder (average particle size: 24 μm, density: 2.2 g·cm^−3^, from dongfu, Shanghai, China); Al powder (average particle size: 24 μm, density: 2.78 g·cm^−3^, from Xingrongyuan, Beijing, China); and Bi_2_O_3_ powder (average particle size: 75 μm/150 μm, density: 8.9 g·cm^−3^, from Xingrongyuan, Beijing, China). The physical and chemical properties of each component are listed in Table 1.

PTFE and Bi_2_O_3_ can undergo intense oxidation-reduction reaction with Al, respectively. The chemical reaction equations are shown in Equations (1) and (2).

(1) $${4\mathrm{Al}\;+\;3C}_{2}F_{4}{\;=\;4\mathrm{AlF}}_{3}\;+\;6C$$

(2) $${2\mathrm{Al}\;+\;\mathrm{Bi}}_{2}O_{3}{\;=\;\mathrm{Al}}_{2}O_{3}\;+\;3\mathrm{Bi}$$

According to the above reaction equations, the stoichiometry ratio of PTFE/Al is 73.5:26.5, and the stoichiometry ratio of Al/Bi_2_O_3_ is 10.96:89.04. Considering the reaction rate, this work prepared the material formulation with PTFE/Al and Al/Bi_2_O_3_ as independent units. PTFE/Al and Al/Bi_2_O_3_ were mixed in different proportions to form material with different formulations. A series of samples were prepared, whose information is listed in Table 2.

The preparation process mainly includes mixing, cold isostatic pressing, and high-temperature sintering. Firstly, the PTFE, Al, and Bi_2_O_3_ raw powders of a certain mass were added to the anhydrous ethanol solution and mixed by a blender for about 40 min, followed by a drying process at room temperature lasting 48 h. Then the mixed powder was placed in a mold with an inner diameter of 10 mm and cold uniaxial pressed at about 250 MPa. Finally, semi-finished samples were placed in a vacuum sintering oven. The oven temperature was raised to 370 °C at a rate of 60 °C/h, then holding at 370 °C for 4.5 h, and cooling to ambient temperature at a rate of 60 °C/h. In the high-temperature sintering process for a long time, PTFE melts and presents a viscoelastic state with a certain degree of fluidity. The polymer chain flows inside the sample to fill the pore formed in the preparation of the sample and improves its compactness. During the cooling process, PTFE recrystallization changes the state of the PTFE matrix in the sample from distributed distribution to integral distribution, which increases PTFE’s bonding performance and improves the overall strength of the sample.

**Figure 1:**
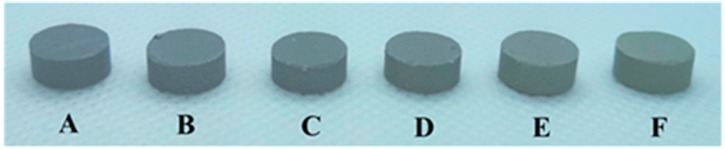
Typical samples with different content: The content of Al/Bi_2_O_3_ is (**A**) 0, (**B**) 5%, (**C**) 10%, (**D**) 20%, (**E**) 30% and (**F**) 40%, respectively.

As shown in Table 2, with the increase of the content of bismuth oxide, the theoretical density of PTFE/Al/Bi_2_O_3_ material theory increases and the energy contained in the unit mass theory decreases. Typically prepared samples (Φ 10 mm × 4 mm) with different formulations are shown in Figure 1. As shown in Figure 1, with the content of Bi_2_O_3_ increasing, the color of the sample gradually changed from gray to gray-green.

### 2.2. Experimental Contents

**Figure 2:**
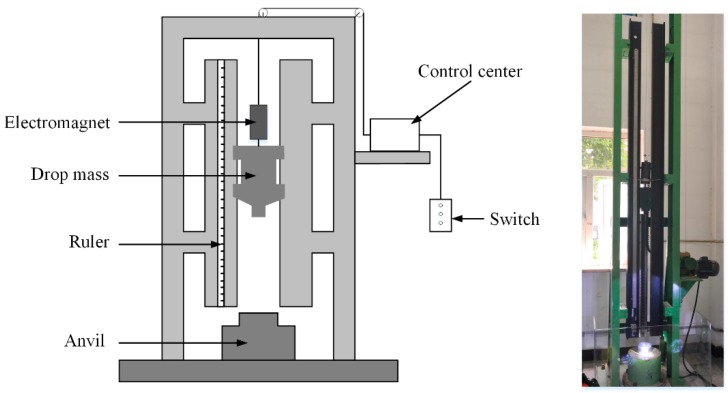
Schematic view and photo of drop-weight test.

The energy release performance of PTFE/Al/Bi_2_O_3_ composites were investigated by a drop-weight device, as shown in Figure 2. In the drop-weight experiment, the test sample is placed in the center of the anvil and directly under the drop-weight. The drop-weight falls freely from a certain height *H* guided by two rods to ensure a planar impact. The test sample is compressed and reacts under the action of the compression load. The drop-weight (mass of 10 kg) carries the upper anvil drops from a height of up to 2 m. The impact sensitivities of each type are compared by the characteristic drop height (*H*_50_), at which the material has a 50% probability of reaction. The test method adopts the well-known “up-and-down technique” [15]. The tests for each type of material were performed 28 times and at 5 cm intervals. In order to study the effect of Bi_2_O_3_ on energy release performance, including reaction intensity, ignition delay time, and reaction duration, the drop-weight was dropped from the same height to impact the materials.

In order to record the sequences of the sample reaction behaviors in the drop-weight test, a Phantom V710 high-speed photography (Vision Research, Inc., Wayne, NJ, USA) was employed. The selected frame rate was 20,000 per second, so that a frame was taken every 50 μs. The resolution was 640 × 480 pixels and the exposure time was set to 10 μs. These settings were selected based on early testing and represent an optimal tradeoff between available lighting and the minimization of blur in the images.

The X-ray diffractometer (XRD, BRUKER D8 ADVANCE, Bruker*,* Karlsruhe Germany) was used to detect the reacted sample after the drop-weight test to analyze the reaction product of PTFE/Al/Bi_2_O_3_. The instrument parameters settings were as follows: the tube voltage was 40 Kv; the current was 40 Ma; Cu K_α_ radiation (λ = 0.15406 nm) was selected; the scanning range 2*θ* was 10°–90°; and the scanning speed was 5° min^−1^.

## 3. Results and Discussion

### 3.1. Mesoscale Characteristics

**Figure 3:**
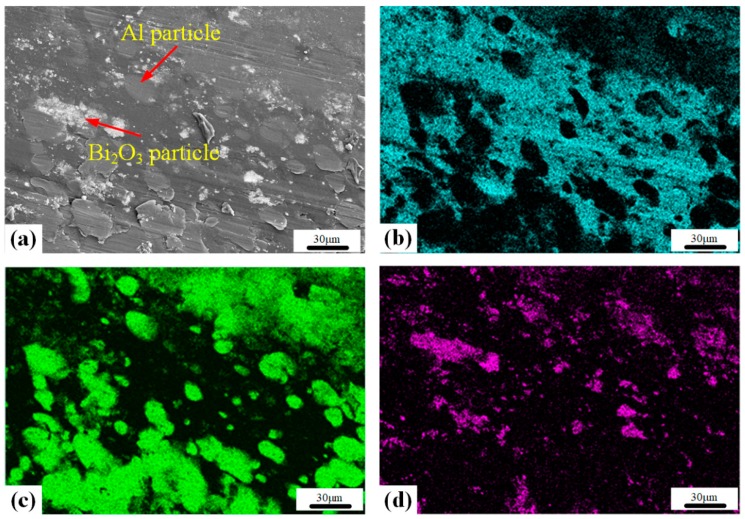
Microstructures and element distribution of the samples: (**a**) Type D-1; (**b**) F element; (**c**) Al element; (**d**) Bi element.

In order to study the mesoscale characteristics of PTFE/Al/Bi_2_O_3_ composite material, scanning electron microscope (SEM, HITACHI S-4800, CamScan, Tokyo, Japan) was used. The mesoscale characteristics are shown in Figure 3. Figure 3a indicates that in the PTFE/Al/Bi_2_O_3_ composite, the PTFE matrix closely wrapped the Al and Bi_2_O_3_ particles. Figure 3b–d show the distribution of elements in the sample. F, Al, and Bi elements represent the distribution of PTFE matrix, Al, and Bi_2_O_3_ in the sample, respectively. The distribution of elements also shows that the Al and Bi_2_O_3_ particles are uniformly distributed in the PTFE matrix and the particle and matrix are tightly bound.

**Figure 4:**
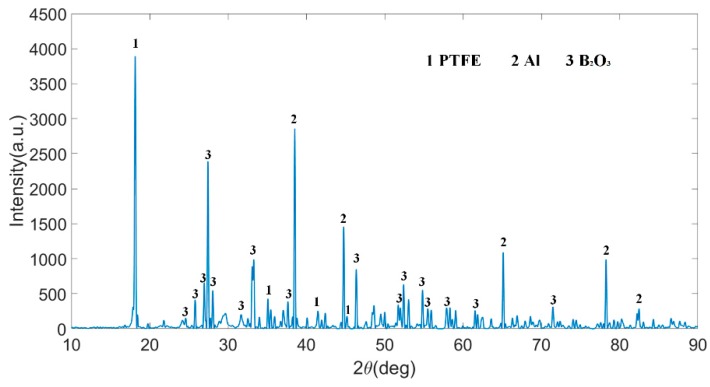
XRD pattern of PTFE/Al/Bi_2_O_3_ (Type D-1).

Figure 4 presents the XRD pattern of PTFE/Al/Bi_2_O_3_ samples (Type D-1). As shown in Figure 4, it indicates that PTFE, Al, and Bi_2_O_3_ existed in the samples, and no new substances had formed. It means that no chemical reaction occurred in the sample during mixing and sintering, and only physical mixing occurred among the components. The components within the PTFE/Al/Bi_2_O_3_ composite still retained their original physical and chemical properties. Therefore, the physical and chemical properties of composite materials can be estimated from the properties of three components. Take the burning rate as an example, according to the superposition principle of burning rate of multi-component materials, the burning rate of Type F is about 253.6 m/s, about 127 times of that of Type A [13].

### 3.2. Impact Sensitivity

**Figure 5:**
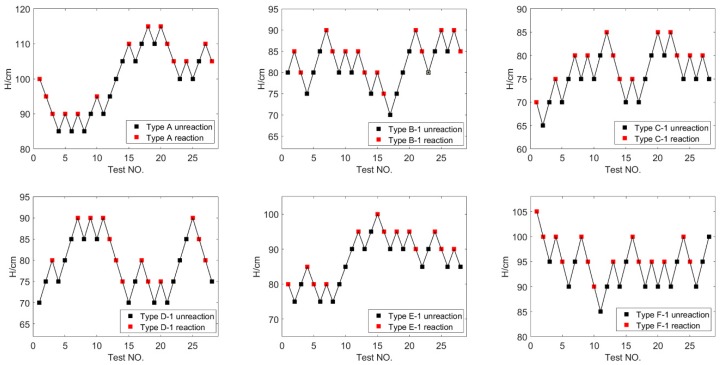
The drop-weight experiments about PTFE/Al/75 μm-Bi_2_O_3_ with different content.

**Figure 6:**
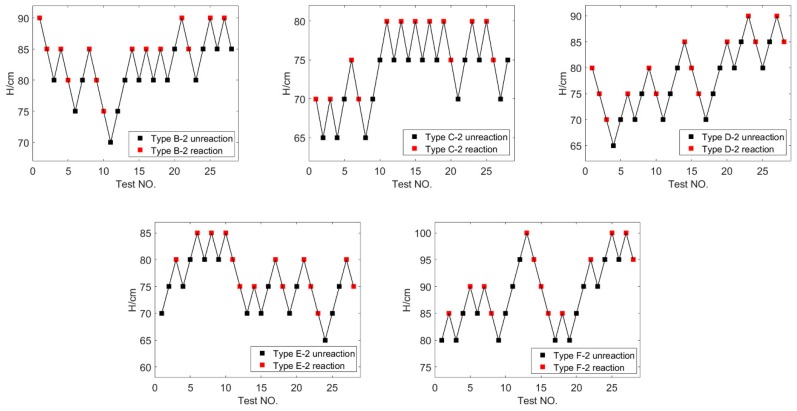
The drop-weight experiments about PTFE/Al/150 μm-Bi_2_O_3_ with different content.

The impact sensitivity of PTFE/Al/Bi_2_O_3_ materials with different content was studied by the drop-weight test and compared by *H*_50_. The drop-weight experiments were recorded as shown in Figure 5 and Figure 6. As shown in Figure 5 and Figure 6, samples may react when impacted by drop mass falling from a range of height. Among them, the range of falling height appeared in the experiment was as wide as 30 cm for Type A, while the falling height range of other types was all about 20 cm, which reflects that the critical reaction height of Type A under impact loading were relatively fuzzy.

**Figure 7:**
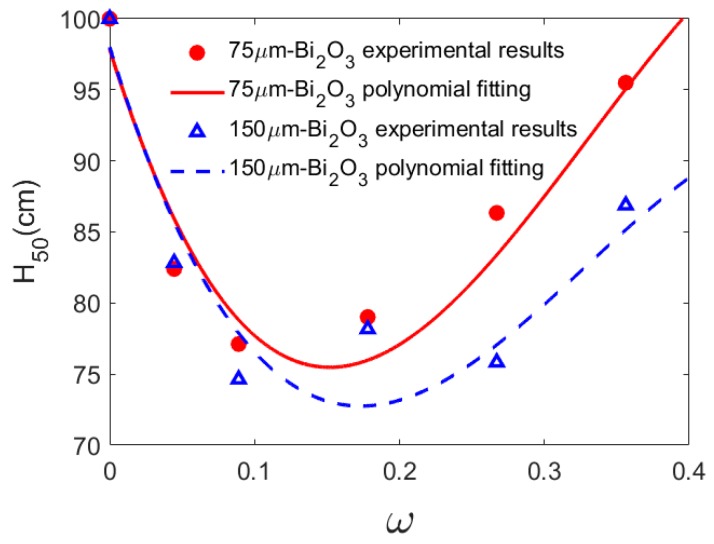
The variation trend of *H*_50_ of PTFE/Al/Bi_2_O_3_ with the content of Bi_2_O_3._

According to the above drop-weight test results, *H*_50_ of each type can be calculated as the following Equation:

(3) $$H_{50}=\;(A+B(\frac{\sum iC_{i}}{D}-\frac{1}{2}))$$

where *A* is the lowest height in the test; *B* is the increment of height, *B* = 5 cm in this test; *D* is the number of reactions that occur in the test; *i* is the ordinal number that falls from low to high, starting from 0; and *C_i_* is the number of times that the samples react at the certain height. By Equation (3), the *H*_50_ of each type is listed in Table 3. The variation trend of *H*_50_ of PTFE/Al/Bi_2_O_3_ with the content of Bi_2_O_3_ is shown in Figure 7.

The results show that the content of Bi_2_O_3_ has a significant influence on the impact sensitivity of PTFE/Al/Bi_2_O_3_ composites. Type A composite has the highest *H*_50_ reaching 100 cm, compared with composite adding the content of Bi_2_O_3_ from 4.452% to 35.616%. In detail, the *H*_50_ of PTFE/Al/Bi_2_O_3_ composite adding 75 μm-Bi_2_O_3_ decreases with the increase of the content of Bi_2_O_3_ ranging from 0% to 17.808%, while the *H*_50_ of the composite increases with the increase of the content of Bi_2_O_3_ ranging from 17.808% to 35.616%. The Type C-1 composite has the highest impact sensitivity, and the *H*_50_ of Type C-1 only 0.77 times of that of Type A. When the particle size of Bi_2_O_3_ is 150 μm, the *H*_50_ varies with the Bi_2_O_3_ content, which is similar to that of Bi_2_O_3_ particle size 75 μm.

The polynomial fitting result of *H*_50_ with the change of content in Figure 7 shows that the particle size of Bi_2_O_3_ has no fundamental influence on the general trend of *H*_50_ with the change of content, but it has significant influence on the change rate of *H*_50_ with the change of content, especially when the content of Bi_2_O_3_ is higher than about 20%. It is worth noting that the minimum *H*_50_ of PTFE/Al/ Bi_2_O_3_ with large particle size Bi_2_O_3_ is lower than that with small particle size Bi_2_O_3_. The reasons may be that Bi_2_O_3_ particle size affects the failure behavior of PTFE/Al/Bi_2_O_3_ composites under the compression, thus affecting the reaction performance of the composites.

The material is subjected to strong compression and failure, and the particles rub against each other, forming ‘hot spots’. The addition of Bi_2_O_3_ in the PTFE/Al system affects the impact sensitivity mainly from the following two aspects. As the content of Bi_2_O_3_ increases, on the one hand, the number of hot spots in the material increases, making the reaction easier to be triggered, leading to the material impact sensitivity increase. On the other hand, in the PTFE/Al/Bi_2_O_3_, the contact area of active PTFE/Al may be reduced due to the separation caused by Bi_2_O_3_ particles, and a portion of the impact energy is transferred to the Bi_2_O_3_ particles, leading to the material impact sensitivity reduction.

### 3.3. Prediction Model for PTFE-Based Reactive Material Impact Sensitivity

In order to analyze the influence of Bi_2_O_3_ content on impact sensitivity of PTFE-based reactive material, the model based on the ‘hot spots’ theory, is presented to predict the *H*_50_ of materials. Two reactions, PTFE/Al and Al/Bi_2_O_3_, occurred in the PTFE/Al/Bi_2_O_3_composite material. In order to simplify the analysis, the following assumptions are made: (1) all particles in the material are distributed randomly and uniformly and tightly packed by the PTFE matrix without pores; (2) particles are spherical with average size.

As analyzed in Section 3.2, the content of Bi_2_O_3_ affects the impact sensitivity of materials from two aspects: (1) as the number of Bi_2_O_3_ particle increases, the number of hot spots formed by slide friction increases, which benefits to induce reaction; (2) as proportion of Al/Bi_2_O_3_ reactants increases, the reaction threshold of PTFE/Al/Bi_2_O_3_ composite increases, which impedes the reaction.

When the hot spots formed by interparticle slip is the dominant mechanism of induced reaction, the following three conditions are necessary: (1) the influence range of the friction hot spots includes the reactants; (2) the reaction temperature threshold is lower than the melting point of the friction particles; (3) the temperature of hot spot is higher than reaction threshold temperature. If all conditions are met, the material will be ignited. It is worth noting that due to the small size of the hot spot, the influence range of hot spot temperature is the particles involved in the friction and PTFE matrix. Therefore, the concept of effective hot spot is proposed, which refers to the hot spot that can induce the reaction. In PTFE/Al/Bi_2_O_3_ composite material, effective friction hot spot refers to the hot spot formed by friction between Al and Al and Bi_2_O_3_, respectively. This is because the melting point of Al is only 933 K below the reaction threshold of Al and Bi_2_O_3_. Hot spots only could induce the reaction between Al and PTFE.

The temperature rise due to slide friction between particles is expressed as [16]

(4) $$\Delta T=\frac{\mu Lu\pi^{1/2}}{4a^{1/2}(k_{1}+k_{2})}$$

where ΔT is temperature rise caused by friction between particles, $k_{1}$ and $k_{2}$ are thermal conductivity of two particles, *a* is the radius of actual contact area, *μ* is the coefficient of friction, *L* is normal pressure, and *u* is relative velocity, respectively.

It assumes that the average relative velocity between particles is equal to half of the drop mass velocity in the drop-weight test. Then, *u* when the drop height is *H*_50_ can be expressed:

(5) $$u=\frac{\sqrt{2gH_{50}}}{2}$$

where g is the gravitational acceleration which is 9.8 m·s^–2^. The friction between two particles during mechanical impact belongs to a random independent event, and the friction probability of Al/Al and Al/Bi_2_O_3_ fraction can be expressed as $v_{\mathrm{Al}}^{2}$ and $2v_{\mathrm{Al}}v_{{\mathrm{Bi}}_{2}O_{3}}$ by the volume fraction, respectively. Considering the uneven temperature and proportion of two-type hot spots, the weighted average temperature rises because of hot spots, and can be approximately expressed as

(6) $$\Delta T_{f}=v_{\mathrm{Al}}^{2}\cdot \Delta T_{\mathrm{Al}/\mathrm{Al}}+2v_{\mathrm{Al}}v_{{\mathrm{Bi}}_{2}O_{3}}\cdot \Delta T_{{\mathrm{Al}/\mathrm{Bi}}_{2}O_{3}}$$

where $v_{\mathrm{Al}}$ and $v_{Bi_{2}O_{3}}$ are volume fraction of Al and Bi_2_O_3_, respectively. $\Delta T_{f}$ is temperature rise. Assume the temperature rise caused by adiabatic shear and heating at creak tips mechanism is *T*_other_, ignoring variation because of Bi_2_O_3_ content, the hot spot temperature of the material under the action of a drop mass can be expressed as

(7) $$T=\frac{\mu Lu\pi^{1/2}}{4a^{1/2}}(\frac{v_{\mathrm{Al}}^{2}}{2k_{\mathrm{Al}}}+\frac{2v_{\mathrm{Al}}v_{{\mathrm{Bi}}_{2}O_{3}}}{k_{\mathrm{Al}}+k_{{\mathrm{Bi}}_{2}O_{3}}})+T_{0}+T_{other}$$

where *T*, *T*_0_ are ultimate and initial temperature of hot spot, respectively. $k_{\mathrm{Al}}$ and $k_{{\mathrm{Bi}}_{2}O_{3}}$ are thermal conductivity of Al and Bi_2_O_3_, respectively.

Hot spot induced material reaction is not only related to hot spot temperature but also affected by hot spot size. The size of hot spots strongly depends on the chemical reaction rate. When the reaction rate *k* is higher than the critical reaction rate *k*_c_, the hot spot size satisfies the starting condition. According to Arrhenius formula, the rate of the chemical reaction can be expressed as

(8) $$k=Ae^{-\frac{E_{a}}{RT}}$$

where *A* is the pre-exponential factor; *E_a_* is the active energy; R is the gas content; and *T* is the temperature, respectively. When $k>k_{c}$, that is to say, the hot spot size reaches the reaction condition, which is reflected as the chemical reaction temperature threshold at the macro level. The reaction temperature threshold can be expressed as

(9) $$T>\frac{1}{-\frac{R}{E_{a}}\ln\frac{k_{c}}{A}}$$

Since constants (*A*, *E*_a_, and *k*_c_) are affected by multiple factors, such as particle size and shape, it is difficult to get detailed data, so this paper directly introduces the reaction temperature threshold to consider the judgment of reaction parameters (*A*, *E*_a_, and *k*_c_) on whether the reaction occurs or not. When PTFE-based granule composites are impacted, the particles within the materials are randomly rubbed against each other. Therefore, the reaction threshold temperature of PTFE/Al/Bi_2_O_3_ composite with different Bi_2_O_3_ content can be estimated as

(10) $$T_{c}=\omega_{{\mathrm{Al}/\mathrm{Bi}}_{2}O_{3}}^{2}T_{{\mathrm{Al}/\mathrm{Bi}}_{2}O_{3}}+(1-\omega_{{\mathrm{Al}/\mathrm{Bi}}_{2}O_{3}}^{2})T_{\mathrm{Al}/\mathrm{PTFE}}$$

where *T*_c_ is the reaction threshold temperature of composite material and $\omega_{{\mathrm{Al}/\mathrm{Bi}}_{2}O_{3}}$ and $\omega_{\mathrm{PTFE}/\mathrm{Al}}$ are the mass fraction of the reactant Al/Bi_2_O_3_, and PTFE/Al, respectively. $T_{\mathrm{Al}/\mathrm{PTFE}}$ and $T_{{\mathrm{Al}/\mathrm{Bi}}_{2}O_{3}}$ are the reaction threshold temperature of PTFE/Al and Al/ Bi_2_O_3_, respectively. When *T* ≥ *T*_c_, the material reaction is triggered. Combined with Equations (5), (7), and (10), the *H*_50_ of PTFE/Al/Bi_2_O_3_ composite material with the content of Bi_2_O_3_ particles can be expressed as

(11) $$H_{50}=\frac{32a}{g\pi \mu^{2}L^{2}}{\left[\frac{\omega_{{\mathrm{Al}/\mathrm{Bi}}_{2}O_{3}}^{2}T_{{\mathrm{Al}/\mathrm{Bi}}_{2}O_{3}}+(1-\omega_{{\mathrm{Al}/\mathrm{Bi}}_{2}O_{3}}^{2})T_{\mathrm{Al}/\mathrm{PTFE}}-T_{0}-T_{other}}{\frac{v_{Al}^{2}}{2k_{Al}}+\frac{2v_{Al}v_{Bi_{2}O_{3}}}{k_{Al}+k_{Bi_{2}O_{3}}}}\right]}^{2}$$

**Figure 8:**
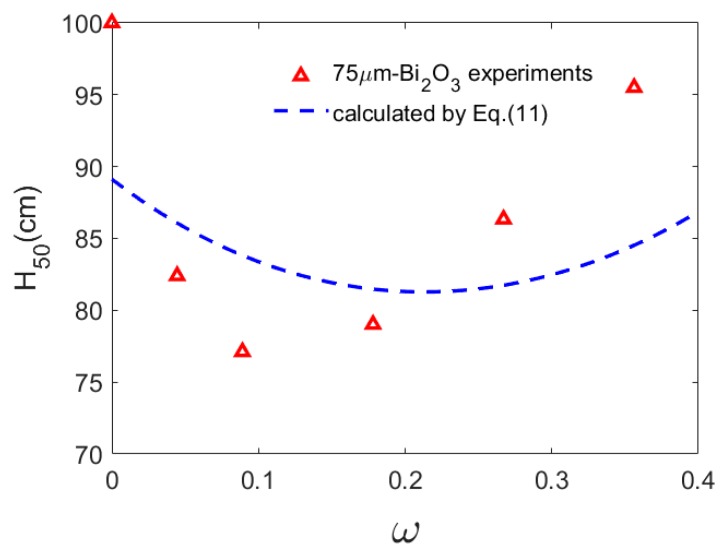
*H*_50_ varies with the content of Bi_2_O_3._

The parameters used in the model are listed in Table 4 [17]. Figure 8 depicts the calculated results of Equation (11).

The model analysis shows that when the content of Bi_2_O_3_ is low, the number and temperature of hot spots dominate *H*_50_ of the PTFE/Al/Bi_2_O_3_ composite materials, leading to the decline of the *H*_50_ with the increase of Bi_2_O_3_ content. When the content of Bi_2_O_3_ is high, the ignition threshold dominates *H*_50_, leading to the improvement of *H*_50_ with the increase of Bi_2_O_3_ content.

On the whole, the above model can predict the change law of *H*_50_ with Bi_2_O_3_ content, but compared with the experimental value, the model predicts the slow change rate of *H*_50_ with Bi_2_O_3_ content, which is because the model only considers the influence of friction hot spots and ignores the change of temperature rise under the influence of content induced by other mechanisms. When the Bi_2_O_3_ content was less than 5%, the model predict lower than the experimental results. The main reason is that friction is not the dominant mechanism of hot spot formation in the low particle content composite material. As PTFE matrix content increases, the material shows good toughness, and the temperature rise caused by adiabatic shear and heating at the crack tip is underestimated, so the prediction results of the model are lower than the experimental results. When the Bi_2_O_3_ content is in the range of 5%–30%, the model prediction is higher than the experimental results. According to the mechanical characteristic analysis of other PTFE/Al materials, the failure strain of the composite material in the range of particle content increases to the maximum and then declines, and the failure strain affects the exothermic change of the crack tip, leading to the prediction model error. When the Bi_2_O_3_ content is higher than about 30%, the model prediction is slightly better than the experimental composite, because friction contributes more to the formation of hot spots in the composite with high particle content.

### 3.4. Energy Release Performance under a Certain Impact

**Figure 9:**
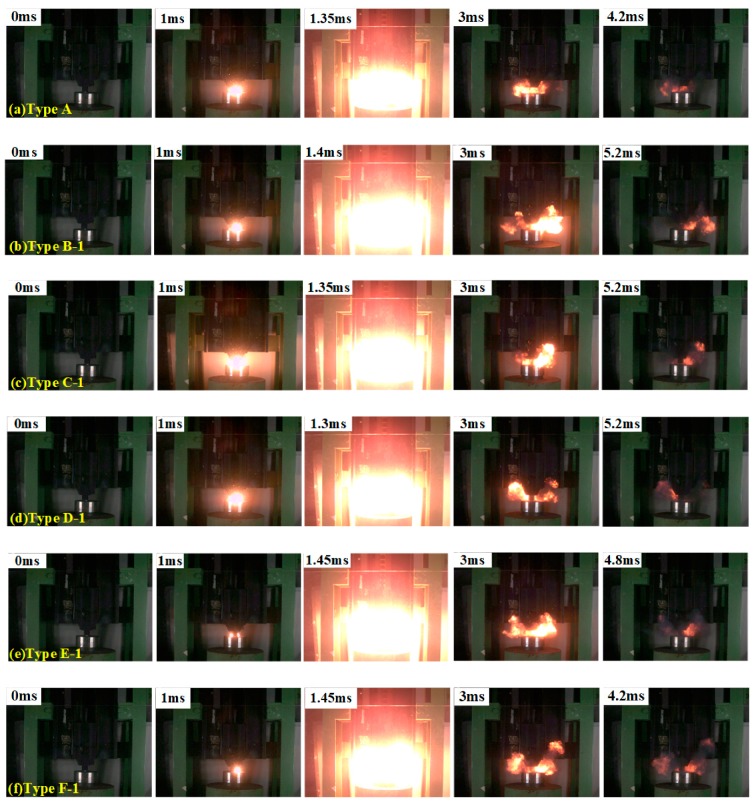
Reaction phenomena of composite materials with 75 μm-Bi_2_O_3_: from left to right, the second frame of each row is the moment of ignition, and the third frame of each row is the moment of the most intense reaction.

**Figure 10:**
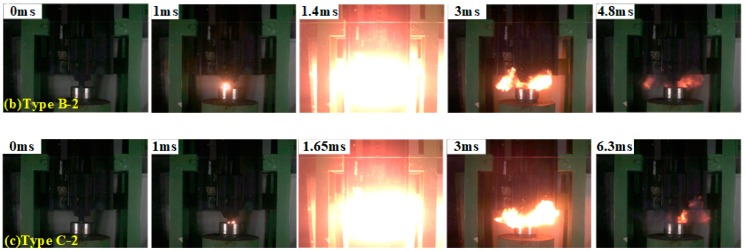

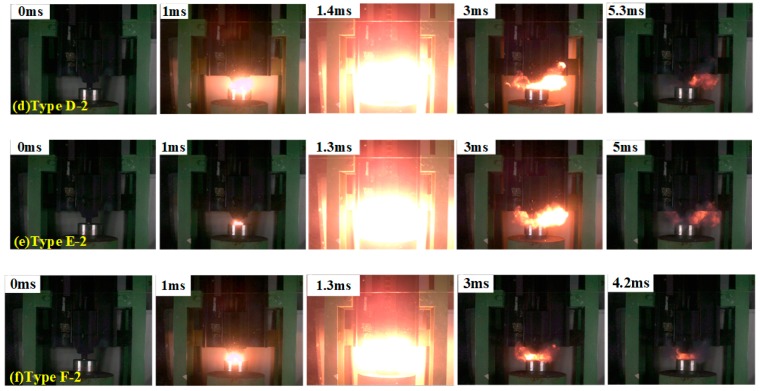
Reaction phenomena of composite materials with 150 μm-Bi_2_O_3_: from left to right, the second frame of each row is the moment of ignition, and the third frame of each row is the moment of the most intense reaction.

Figure 9 and Figure 10 displays the reaction phenomena of composite materials with different Bi_2_O_3_ content under a certain impact. As shown in the Figure 9 and Figure 10, when the samples are compressed by a drop-weight with a fall height of 140 cm, the samples all react violently, and the reaction intensity strongly depends on the content of Bi_2_O_3_.

The moment of contact between the drop-weight and the sample is 0. The samples are intensely compressed first, after about 1.3 to 1.65 ms reaction starting. These results imply that the content and particle size of Bi_2_O_3_ have no significant effect on the delay time of composite materials. Type A composite has the lowest reaction intensity, compared with PTFE/Al composite material adding 4.452% to 35.616% Bi_2_O_3_. With the increase of Bi_2_O_3_ content, the reaction degree increases first, and then reduces. The most violent reaction of PTFE/Al/Bi_2_O_3_ occurs at the Bi_2_O_3_ content of 4.452% to 8.904%. The experiments indicate that PTFE/Al/Bi_2_O_3_ with proper Bi_2_O_3_ content, which is called optimum content of Bi_2_O_3_, could maximize the reaction degree of the PTFE/Al/Bi_2_O_3_ composite.

The content of Bi_2_O_3_ has a significant influence on the reaction duration. The reaction duration of the sample without Bi_2_O_3_ (Type A) is about 4.2 ms. In general, as the content of Bi_2_O_3_ increases, the reaction duration is prolonged; while, when the content of Bi_2_O_3_ exceeds a certain value, the reaction duration is shortened. When the particle size of Bi_2_O_3_ is 75 μm, there is no significant difference in reaction duration (about 5.2 ms) in range from 4.452% to 17.808%. When the content exceeds 17.808%, the reaction duration shortens. For the samples with 150 μm-Bi_2_O_3_, when the content of Bi_2_O_3_ is 8.904%, the reaction duration reaches about 6.3 ms at most. As the content of Bi_2_O_3_ continues to increase, the reaction duration of samples shortens. It can be illustrated from reactions, which occur in the PTFE/Al/Bi_2_O_3_ composite materials, PTFE/Al reacts at a lower temperature, but the burning rate of PTFE/Al is slow and the reaction has significant non-self-sustainability. Al/Bi_2_O_3_ reacts at a higher critical temperature, comparing with the PTFE/Al, and the burning rate of Al/Bi_2_O_3_ is faster. In the PTFE/Al/Bi_2_O_3_ composite, the PTFE/Al reaction occurs first, and releases a large amount of heat. A large amount of heat will induce the reaction between Al and Bi_2_O_3_. A large amount of heat released by the reaction Al/Bi_2_O_3_ and the high burning rate is conducive to the reaction, improving the self-sustainability of reaction. Thus, the reaction duration of the material is prolonged. However, the increase of Bi_2_O_3_ content means that the content of PTFE/Al decreases, and the heat released by the reaction of PTFE/Al decreases, which is not conducive to induce the secondary reaction of Al/Bi_2_O_3_, leading to more incomplete reaction and shorter reaction duration.

**Figure 11:**
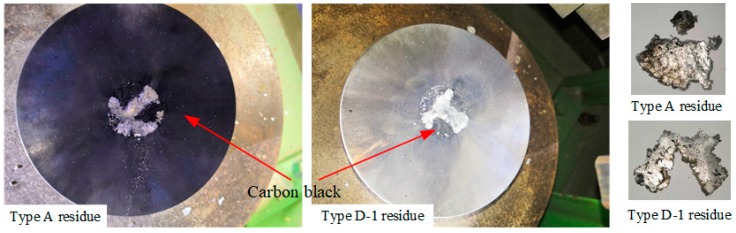
Recovered residues and reaction vestiges.

The state of two samples, Type A (without Bi_2_O_3_) and Type D-1 (with 75 μm-Bi_2_O_3_), after impact, are shown in Figure 11. There is a large amount of carbon black remaining on the anvil after the reaction of Type A sample, while less carbon black after reaction of Type D-1 sample. The amorphous carbon generated by the reaction is mainly distributed on the edge of the residue sample. Besides, carbon also exists on the surface of the Type A sample. The phenomenon indicates that Type D-1 reacts more thoroughly than Type A under the drop mass height of 140 cm. That is probably because Bi_2_O_3_ could react with amorphous carbon of PTFE/Al reaction product at high temperature [18], so the reaction degree is improved and the carbon black reduces. Compared with Type D-1 residue, Type A residue is more complete, which also indicates that the reaction rate of Type A is lower than that of Type D-1, and opportune Bi_2_O_3_ content is conducive to the reaction.

### 3.5. Reaction Mechanism

**Figure 12:**
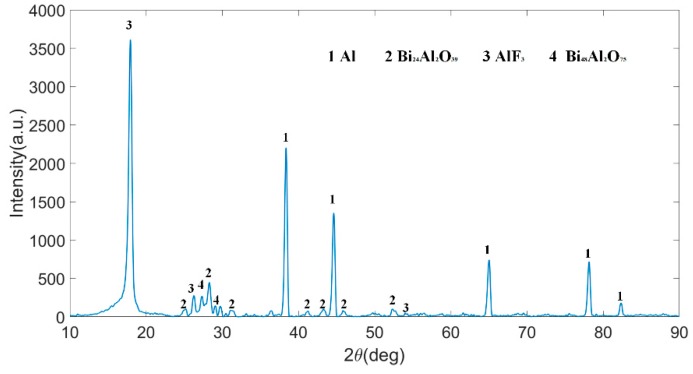
X-ray diffraction pattern of the reaction residue of Type D-1.

In order to understand the chemical reaction mechanism of the PTFE/Al/Bi_2_O_3_ composites, the reacted residue of Type D-1 sample is analyzed by X-ray diffraction (XRD), and the result is shown in Figure 12. The results show that AlF_3_, Bi_24_Al_2_O_39_, and Bi_48_Al_2_O_75_ are produced during the reaction. Among them, AlF_3_ is the product of the reaction between Al and gaseous C_2_F_4_ generated by PTFE cracking. In addition, the reaction also generates amorphous carbon, which could not be detected by XRD. Bi_24_Al_2_O_39_ and Bi_48_Al_2_O_75_ are two kinds of mixed crystals formed by Bi_2_O_3_ and Al_2_O_3_ at high temperature. Bi_2_O_3_ reacts with Al to form Al_2_O_3_ and Bi, and unreacted melted Bi_2_O_3_ (melting point: 1098 K) wraps Al_2_O_3_ to form *x*Bi_2_O_3_·Al_2_O_3_. Bi, the other product of the reaction between Al and Bi_2_O_3_, is also not detected in the product. It is because that, Bi, whose boiling point is only 1833 K, forms Bi vapor due to the high temperature during the reaction process. The formation of *x*Bi_2_O_3_·Al_2_O_3_ crystal also indicates a small Al and Bi_2_O_3_ participation reaction. It implies that the materials react incompletely under the low impact load. The analysis in Section 3.4 also indicates that C and Bi_2_O_3_ may have subsequent reactions. Combining the analysis above, the possible chemical reaction process of the PTFE/Al/Bi_2_O_3_ composite can be described as:

(12) $$\left(-C_{2}F_{4}-\right)\overset{}{\to }C_{2}F_{4}↑$$

(13) $$\mathrm{Al}+C_{2}F_{4}\overset{}{\to }{\mathrm{AlF}}_{3}+C$$

(14) $${\mathrm{Bi}}_{2}O_{3}+\mathrm{Al}\overset{}{\to }{\mathrm{Bi}}_{24}{\mathrm{Al}}_{2}O_{39}+\mathrm{Bi}↑$$

(15) $${\mathrm{Bi}}_{2}O_{3}+\mathrm{Al}\overset{}{\to }{\mathrm{Bi}}_{48}{\mathrm{Al}}_{2}O_{75}+\mathrm{Bi}↑$$

(16) $${\mathrm{Bi}}_{2}O_{3}+C\overset{}{\to }{\mathrm{CO}}_{2}↑+\mathrm{Bi}↑$$

The above analysis shows that when PTFE/Al/Bi_2_O_3_ composite material is impacted, not only does it react with PTFE and Bi_2_O_3_, respectively, with Al as the oxidant, but also PTFE in the composite reacts with Bi as the oxidant; in addition, Bi_2_O_3_ may further oxidize reaction product C. Therefore, Bi_2_O_3_ can effectively improve the energy release characteristics of PTFE/Al-based energetic materials.

**Figure 13:**
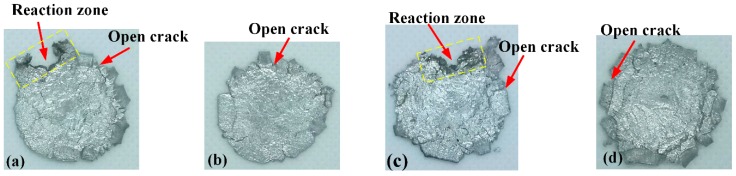
Recovered sample residues from drop-weight tests: (**a**) reacted residues of Type D-1; (**b**) unreacted residues of Type D-1; (**c**) reacted residues of Type D-2; (**d**) unreacted residues of Type D-2.

In general, the PTFE/Al/Bi_2_O_3_, a kind of granular composite reactive materials, would be initiated due to the ‘hot spots’, which are probably formed by the sliding friction, adiabatic shear, and heating at crack tips, during mechanical impact. Figure 13 presents the residue of Type D, including reacted and unreacted samples. There is a remarkable difference between reacted and unreacted sample residue. There are several open cracks in the edge of the reacted residue, some of which have black marks and obvious melted marks (Figure 13a,c). While there are only open cracks at the unreacted residue edge (Figure 13b,d). These phenomena imply that the reaction is most likely to begin at the open crack in the material and that the formation of the crack is related to the material reaction. The black marks show that the sample reacts incompletely. The edge open cracks of Type D-2 residue are more obvious than that of Type D-1, indicating that the ductility of the material adding large particle size Bi_2_O_3_ is weaker than that of adding small particle size Bi_2_O_3_ material (Figure 13b,d).

**Figure 14:**
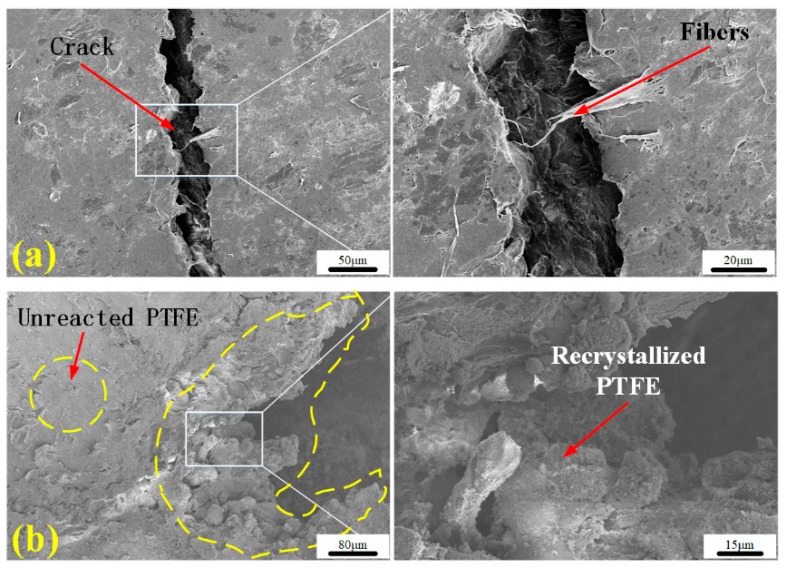
Mesoscale characteristics recovered residues: (**a**) unreacted residue; (**b**) reacted residue.

Figure 14 shows the microscopic characteristics of the cracks in the reacted and unreacted samples. There are no reaction products, such as carbon and bismuth, in shear cracks of unreacted residues. There are fibers, whose direction is perpendicular to that of crack that can be observed clearly, from unreacted residue SEM images. The morphology of crack is similar to that of brittle fracture. Morphology features of unreacted residue are significantly different from those of reacted residue. The crack edge of the sample in which the reaction occurred was irregular and appeared coral-like, which is formed by melted and recrystallization of PTFE in the reaction zone during the reaction. It can be seen from Figure 14b that when the material reacts partially, the reaction zone is generally located at the crack, which also indicates that exothermic heat at the crack tip is an important factor for ignition.

Combined with the results in Section 3.2, it indicates that the mechanism of PTFE/Al/Bi_2_O_3_ composite impact-induced reaction could be described as: (1) under mechanical impact, violent plastic deformation of the material results in the rise of the overall temperature of the material; (2) during the intense compression of granular composite material, violent friction occurs, resulting in hot spots and local temperature rise, which are randomly distributed with in the composite; (3) under the action of strong shear, cracks are formed at the edge of the material, and heat is released from the crack tip, making the crack tip to form hot spots. Considering the above three aspects, the hot spot at the crack tip of the material has the highest temperature and is the easiest to trigger the reaction.

## 4. Conclusions

In this paper, Bi_2_O_3_ was introduced into PTFE/Al reactive composite material. The reaction behaviors of this novel material was studied from energy release performance and reaction mechanism by the drop-weight test, XRD, and SEM. The main conclusions drawn are as follows:

(1) The particles are uniformly distributed in PTFE/Al/Bi_2_O_3_ composite and bonded well with the PTFE matrix. The properties of PTFE/Al/Bi_2_O_3_ can be estimated from the properties of the components.
(2) Bi_2_O_3_ has a significant influence on PTFE/Al/Bi_2_O_3_ composite impact sensitivity. The content of Bi_2_O_3_ increased from 0% to 35.616%, the impact sensitivity of PTFE/Al/Bi_2_O_3_ composite increase first and then decrease, and the lowest *H*_50_ (Type C-2, 10%, 150 μm) was 74.04 cm, 0.74 times that of PTFE/Al. The model for predicting the PTFE-based reactive material impact sensitivity is presented, which is in good agreement with the experimental results.
(3) Energy release performance of PTFE/Al/Bi_2_O_3_ strongly depend on the Bi_2_O_3_ content. At the drop height of 140 cm, as the content of Bi_2_O_3_ increases, the reaction intensity and duration first increases and then decreases. The maximum reaction duration (Type C-2, 10%, 150 μm) is about 6.3 ms, which is 1.5 times that of PTFE/Al.
(4) The main reaction products include AlF_3_, *x*Bi_2_O_3_·Al_2_O_3_, and Bi. In PTFE/Al/Bi_2_O_3_, Bi_2_O_3_, as a reductant, reacts with Al and C, which significantly improves the energy release characteristics of materials. The PTFE in the reaction zone is coral-like, which is significantly different from the morphology of the unreacted zone.

## Figures and Tables

**Table 1:** Summary of element properties of PTFE/Al/Bi_2_O_3._

	*ρ* (g·cm^−3^)	Melting/Boiling Point (K)	Decomposition Point (K)	Morphology	Theoretical Δ*H* of Reaction with Al (kJ/g)
PTFE	2.20	614/-	829	White powder	28.9
Al	2.78	933/2600	-	Silver powder	-
Bi_2_O_3_	8.90	1098/1833	1620	Faint yellow powder	2.36

**Table 2:** Typical samples with different content of PTFE/Al/Bi_2_O_3._

	PTFE/Al ^1^ (wt.%)	Al/75 μm-Bi_2_O_3_^2^ (wt.%)	Al/150 μm-Bi_2_O_3_^2^ (wt.%)	Bi_2_O_3_ (wt.%)	*ρ* (g cm^−3^)	Theoretical Δ*H* (kJ/g)
A	100	0	0	0	2.329	8.420
B-1	95	5	0	4.452	2.414	8.105
B-2	95	0	5		2.414	
C-1	90	10	0	8.904	2.506	7.790
C-2	90	0	10		2.506	
D-1	80	20	0	17.808	2.712	7.159
D-2	80	0	20		2.712	
E-1	70	30	0	26.712	2.955	6.529
E-2	70	0	30		2.955	
F-1	60	40	0	35.616	3.247	5.898
F-2	60	0	40		3.247	

^1^ PTFE/Al: 26.5 wt.%/73.5 wt.%; ^2^ Al/Bi_2_O_3_: 10.96 wt.%/89.04 wt.%.

**Table 3:** Drop height (*H*_50_) of various types of composite materials (cm).

	A	B	C	D	E	F
75 μm	100.00	82.14	76.73	80.19	87.50	94.64
150 μm		82.50	74.04	78.21	76.07	89.42

**Table 4:** The parameters in the model.

$k_{\mathrm{Al}}$ (W m^−1^ K^−1^)	$k_{{\mathrm{Bi}}_{2}O_{3}}$ (W m^−1^ K^−1^)	$T_{\mathrm{Al}/\mathrm{PTFE}}$ (K)	$T_{{\mathrm{Al}/\mathrm{Bi}}_{2}O_{3}}$ (K)	*a* (m)	*μ*	*L* (KN)	*T*_0_ (K)	*T*_other_ (K)
237	900	793	1153	2 × 10^−6^	0.27	17	273	197
